# Heart Block as a Rare Complication of Tricuspid Valve Endocarditis: Awareness is the Key

**DOI:** 10.7759/cureus.22269

**Published:** 2022-02-16

**Authors:** Mrhaf Alsamman, Jing Hao Du, Naser Mubarak, Anamarys Blanco, Kenneth Iyamu

**Affiliations:** 1 Internal Medicine, Health Corporation of America-University of Central Florida (HCA-UCF) Consortium, Ocala, USA; 2 Medicine, University of Central Florida College of Medicine, Orlando, USA

**Keywords:** third-degree heart block, first-degree atrioventricular block, vegetation, septic emboli, tricuspid valve endocarditis

## Abstract

Infective endocarditis (IE) occurs when bacterial or fungal pathogens enter the blood and attach to the endocardium. Right-sided endocarditis is usually associated with intravenous drug use (IVDU), intracardiac devices, and central venous catheters. There is more data published about left-side endocarditis when compared to right-sided endocarditis. Tricuspid valve infective endocarditis (TVIE) accounts for 5%-10% of IE, and of those cases, roughly 10% are complicated by conduction deficits due to inflammatory edema, myocarditis, and abscess formation. Tricuspid valve (TV) surgical repair carries its own risks, one of which includes the development of conduction abnormalities. Here, we review the current data of TVIE complicated by heart block after tricuspid valve replacement. Also, we present a case of a 21-year-old IVDU female who presented with tricuspid valve endocarditis, subsequently underwent tricuspid valve replacement, and developed a heart block.

## Introduction

When a bacterial or fungal pathogen enters the blood and attaches to the endocardium, it is known as infective endocarditis (IE) [1]. There is more data published about left-side endocarditis when compared to right-sided endocarditis. Right-sided IE is often related to intravenous drug use (IVDU), intracardiac devices, and central venous catheters. Between the years 2010 and 2015, the percentage of IVDU in all IE cases increased by 14% with an increase in incidence in younger populations [2]. While intravenous antibiotic remains the mainstay treatment for patients with tricuspid valve infective endocarditis (TVIE), surgery is indicated in recurrent septic pulmonary emboli, large vegetation, failure of medical therapy, and infected prosthetic valves. There is very little research outside small, single-institution case series because the majority of cases are medically handled and only a small proportion of TVIE cases are surgically operated on at each institution [3]. Here, we review the current data of TVIE complicated by heart block after tricuspid valve (TV) replacement. Also, we present a case of a 21-year-old IVDU female who presented with tricuspid valve endocarditis, subsequently underwent tricuspid valve replacement, and developed a heart block.

## Case presentation

**Figure 1:**
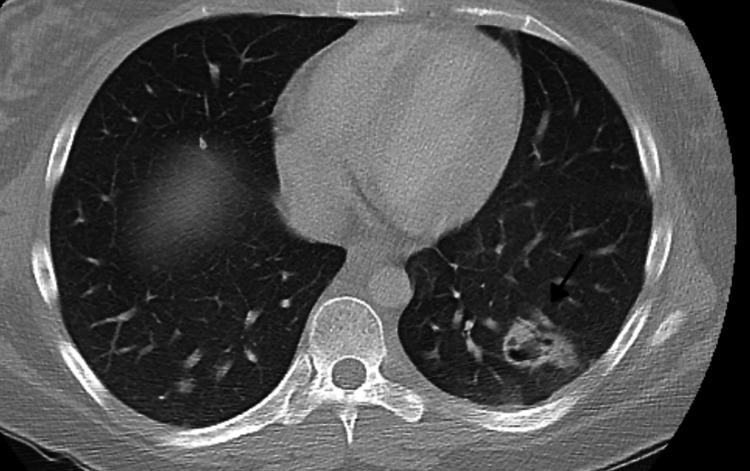
Computed tomography (CT) of the chest Black arrow showing the cavitary lesion measuring approximately 3 cm in diameter

**Figure 2:**
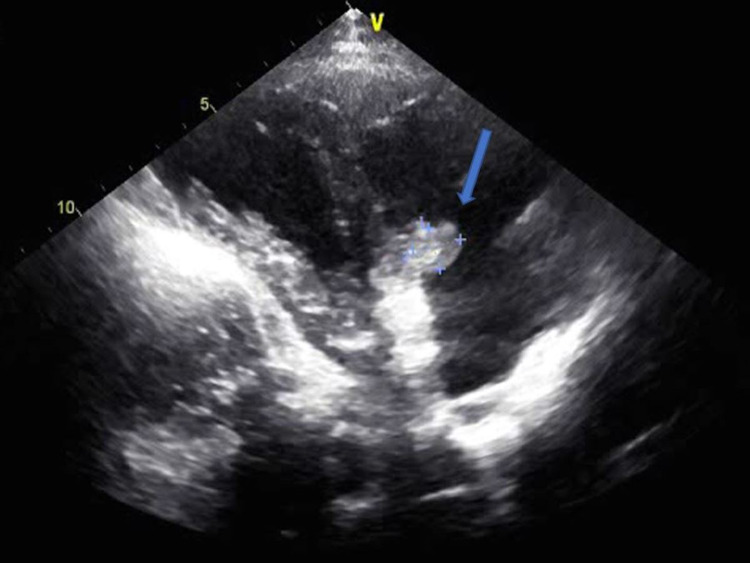
Transthoracic echocardiogram short-axis view Blue arrow showing tricuspid valve vegetation

**Figure 3:**
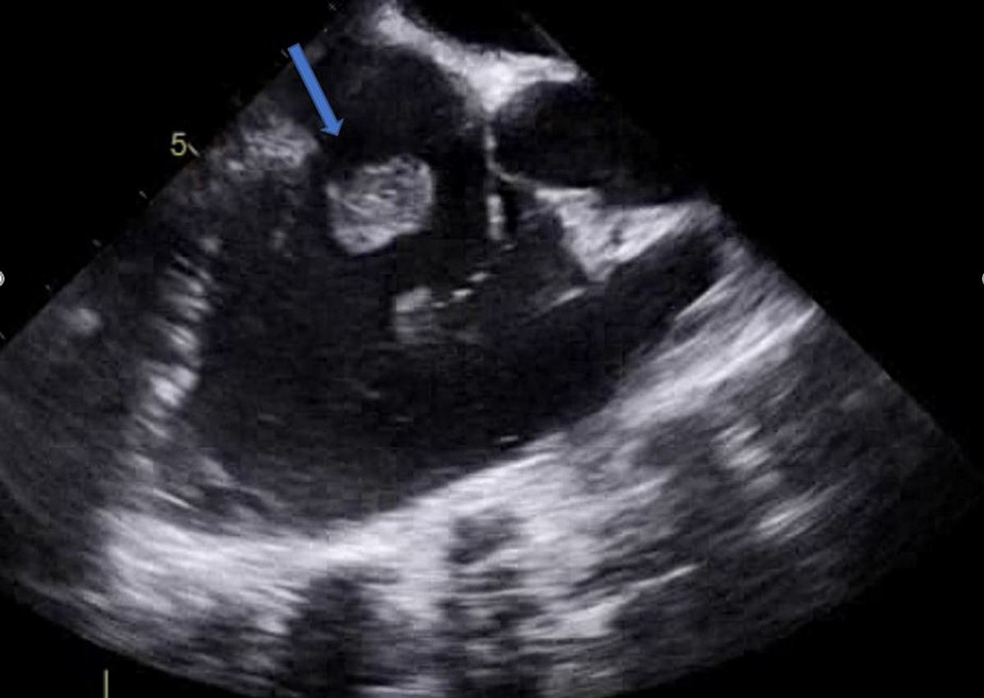
Transesophageal echocardiogram three-chamber mid-esophagus view Blue arrow showing tricuspid valve vegetation

**Figure 4:**
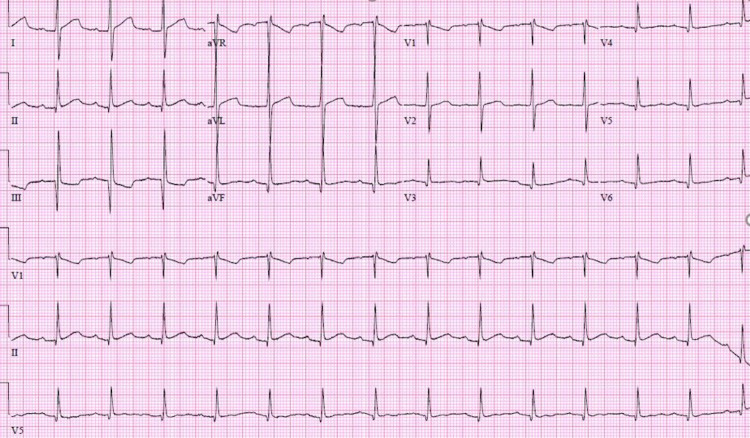
Twelve-lead electrocardiogram demonstrating sinus rhythm with first-degree atrioventricular block, PR interval of 212 ms, ventricular rate of 89 beats per minute, and QRS duration of 82 ms

A 21-year-old female with a significant past medical history of intravenous drug use (fentanyl and methamphetamines) presented to the emergency department with a seven-day history of worsening right-sided neck pain radiating toward her spine and down to both hips. The patient experienced subjective fever and chills, tension headaches, and diaphoresis. Upon admission, her vital signs showed a temperature of 98°F, blood pressure of 107/56 mmHg, pulse rate of 112 beats per minute, respiratory rate of 18 breaths per minute, and oxygen saturation of 98% on room air. Laboratory results revealed thrombocytopenia (platelet count: 45,000/mm^3^), lactic acid of 3.2 mmol/L, creatinine of 0.7 mg/dL, total bilirubin of 0.9 mg/dL, AST of 80 IU/L, ALT of 44 U/L, and inflammatory markers ESR of 46 mm/hour and CRP of 19.1 mg/dL. The patient was subsequently started on vancomycin and cefepime. Computed tomography (CT) of the chest with contrast showed multiple bilateral pulmonary cavitary lesions consistent with the appearance of septic emboli, the largest of which measures approximately 3 cm in diameter (Figure 1). CT of the abdomen and pelvis with contrast is consistent with the findings of pulmonary septic emboli and splenomegaly. Two sets of blood cultures 48 hours apart showed two out of two methicillin-susceptible *Staphylococcus aureus*. The antibiotic regimen was changed to cefazolin. Transthoracic echocardiogram (TTE) and transesophageal echocardiogram (TEE) showed an estimated ejection fraction of 55%, with 1 cm in width × 1.2 cm in length, frond-like, mobile vegetation in the tricuspid valve and mild tricuspid regurgitation (Figure 2 and Figure 3). The patient was evaluated by cardiothoracic surgery and subsequently underwent tricuspid valve replacement with debridement of the right ventricular septum. The patient was then transferred to the intensive care unit (ICU) for postoperative care. During the time in the ICU, the patient developed a complete atrioventricular (AV) block. The complete AV block resolved spontaneously, and the patient converted to a first-degree AV block (Figure 4), which persisted. The course of stay was otherwise uneventful, and she was discharged on intravenous (IV) antibiotics for six weeks.

## Discussion

TV endocarditis accounts for 5%-10% of IE, and of those cases, roughly 10% are complicated by conduction deficits due to myocarditis, inflammation, edema, and abscess formation. This primarily occurs with aortic valve involvement, and to the best of our knowledge, there have only been four previously reported cases of conduction disease due to native TVIE [4-6]. Previous cases reported Mobitz type 2 heart block as a complication of TV endocarditis [4]. Only two cases of extended-spectrum beta-lactamase (ESBL)* Escherichia coli *TV endocarditis resulted in complete AV block [6,7]. The site of the infection is the most important factor in the development of conduction disease. The AV node located in the Koch’s triangle on the septal wall of the right atrium is bounded by the septal leaflet of the TV [7]. The AV node and conduction tissue are located near the junction of the TV’s anterior and septal leaflets, with the aortic valve on the opposite side. Inflammation, edema, and TVIE expansion affecting the septal leaflet may produce changes in the surrounding conduction tissue, which could be a mechanism for TVIE-induced conduction disease. In intravenous drug users, such as our patient, the tricuspid valve is the most commonly affected valve. Transthoracic echocardiography (TTE) and/or transesophageal echocardiogram (TEE), as well as blood cultures, are the standard tests for diagnosis. When TEE and TTE are combined, vegetation may be visible in 90% of the cases [8]. Antibiotic medication is required for all patients with IE, and the length of treatment varies on whether a native or prosthetic valve is involved. The American College of Cardiology (ACC) recommends that surgery be considered in native valve endocarditis with mobile vegetation greater than 10 mm, and the European Society of Cardiology (ESC) concurs [9,10]. Despite the agreement of the ACC and ESC on surgical consideration, there has been no definite consensus on the ideal time for surgery. Heart failure with severe regurgitation is another indication for surgical intervention. In addition, vegetations at high risk of a peripheral embolic event, abscess, or multidrug-resistant infections are also considered for urgent surgical intervention. TV surgical repair carries its own risks, one of which includes the development of conduction abnormalities. One study found that 5.8% of patients required pacemaker implantation one year post TV surgery due to an AV block [11]. As such, it is possible that the AV block seen in our patient may have been secondary to surgical complications or an inducer of conduction pathology secondary to the TV endocarditis.

## Conclusions

We highlighted a case of right-sided endocarditis complicated by heart block following a native tricuspid valve repair. While intravenous antibiotic remains the mainstay treatment, surgery is indicated in large vegetation, recurrent septic pulmonary emboli, failure of medical therapy, and infected prosthetic valves. Surgery carries the risk of developing a heart block due to the anatomical location of the AV node as seen in our patient. Early diagnosis and raising awareness facilitate better outcomes.

## References

[1] Chu VH (2018). Endocarditis. JAMA.

[2] Shmueli H, Thomas F, Flint N, Setia G, Janjic A, Siegel RJ (2020). Right-sided infective endocarditis 2020: challenges and updates in diagnosis and treatment. J Am Heart Assoc.

[3] Hussain ST, Witten J, Shrestha NK, Blackstone EH, Pettersson GB (2017). Tricuspid valve endocarditis. Ann Cardiothorac Surg.

[4] Agu CC, Salhan D, Bakhit A (2015). Tricuspid valve endocarditis complicated by Mobitz type II heart block - a case report and literature review. J Community Hosp Intern Med Perspect.

[5] Martínez-Urueña N, Hernández C, Duro IC, Sandín MG, Zatarain E, San Román A (2012). Transient trifascicular block secondary to tricuspid valve endocarditis. Rev Esp Cardiol (Engl Ed.

[6] Fordyce CB, Leather RA, Partlow E, Swiggum EA (2011). Complete heart block associated with tricuspid valve endocarditis due to extended spectrum β-lactamase-producing Escherichia coli. Can J Cardiol.

[7] Singh N, Kalathiya RJ (2021). Transient complete heart block: a case report of a rare complication of tricuspid valve infective endocarditis. Eur Heart J Case Rep.

[8] Nemati M, Galang K, Jung SM (2020). Right and left-sided infective endocarditis in an IV drug abuser. J Community Hosp Intern Med Perspect.

[9] Murdoch DR, Corey GR, Hoen B (2009). Clinical presentation, etiology, and outcome of infective endocarditis in the 21st century: the International Collaboration on Endocarditis-Prospective Cohort Study. Arch Intern Med.

[10] Nishimura RA, Otto CM, Bonow RO (2014). 2014 AHA/ACC Guideline for the Management of Patients With Valvular Heart Disease: executive summary: a report of the American College of Cardiology/American Heart Association Task Force on Practice Guidelines. Circulation.

[11] Atallah PC (2013). Significance of first-degree atrioventricular block in acute endocarditis. JAMA Intern Med.

